# Gastrointestinal Cancer Metastasis

**DOI:** 10.1155/2012/415498

**Published:** 2012-12-22

**Authors:** Jin-Lian Chen, Richard Ricachenevsky Gurski, Keiichi Takahashi, Roland Andersson

**Affiliations:** ^1^Department of Gastroenterology, School of Medicine, Shanghai East Hospital, Tongji University, Shanghai 200120, China; ^2^Division of Digestive Surgery, Department of General Surgery, Medical School, Hospital de Clínicas de Porto Alegre, Federal University of Rio Grande do Sul, Porto Alegre, RS, Brazil; ^3^Department of Surgery, Tokyo Metropolitan Cancer and Infectious Diseases Center, Komagome Hospital, 3-18-22 Honkomagome, Bunkyo-ku, Tokyo 113-8677, Japan; ^4^Department of Surgery, Clinical Sciences, Lund, Skåne University Hospital and Lund University, 22185 Lund, Sweden

It is well acknowledged that the poor prognosis of gastrointestinal cancer is largely due to metastasis, which is the biological hallmark of malignant tumors. Metastasis is considered to be a multistep process in which malignant tumor cells spread inconsecutively from original site to distant organs. As for cancers of gastrointestinal tract, the common target site for metastasis is liver, lymph nodes, peritoneal, and, subsequently, lung and other sites of the body, which in single or as a whole, is the major cause of gastrointestinal cancer-related deaths.

Therefore, the early diagnosis and effective treatment of gastrointestinal cancer metastasis is of vital importance, which may lead to substantial elongation of life expectancy of gastrointestinal cancer patients. In order to make further progressions in early diagnosis and intervention, the following three directions should be put due emphases on in future research. 

Firstly, the cellular and molecular mechanisms of gastrointestinal cancer metastasis should be clarified, such as the mechanisms of local angiogenesis, the heterogeneity of cancer cells, the cross-talk and cascade of signaling transduction systems, the decreased expression of cell adhesion molecules such as E-cadherin, and so on. To elucidate these mechanisms and to block relevant molecular pathways provides the most promising perspectives in the fight against gastrointestinal caner metastasis. And additionally, there are several interesting and enlightening hot points: the relationship between stromal microenvironment and metastatic destination; the role of cancer stem cells in metastasis; and how malignant cells get metastasis ability, inherent or acquired?

The second direction is to find out specific and sensible biomarkers of gastrointestinal cancer metastasis and to predict the potential metastasis, to facilitate early diagnosis, which is up to now mainly dependent on insensitive and costly radiographic examinations. And the third one is the optimization of comprehensive treatment including surgical intervention.

In this special issue, eight articles on gastrointestinal cancer metastasis are presented. One article discussed the advantages and disadvantages of repeated liver resection in patients with recurrent colorectal liver metastasis. One article reported that N-desulfated heparin is capable of suppressing the metastasis of gastric cancer through inhibiting tumor bFGF expression and tumor angiogenesis with no obvious anticoagulant activity. Yet another article is on the relevance between dietary salt intake and the risk of gastric cancer. The other five articles highlighted four kinds of potential biomarkers for gastrointestinal cancer screening and early diagnosis of metastasis, namely urine-free amino acid, ki-67 antigen, mir-21 and epidermal growth factor receptor (EGFR). J. Fan and his colleagues, through analyzing amino acids in urine samples of gastric cancer patients and healthy volunteers, figured out that urine-free amino acid profiling is of potential value for screening or diagnosing gastric cancer. D. Navarini and his colleagues, from Brazil, reviewed thirty-seven patients who underwent esophagectomy without presurgical chemotherapy or radiotherapy from 2000 to 2010, analyzed the level of EGFR expression and life span, and concluded that EGFR expression is related to higher TNM staging and shorter survival.

Despite the obvious advancements made in the research of gastrointestinal cancer metastasis these years, the outcomes are far from final satisfaction, and thus it is still a field that deserves and calls for intensive and thorough research in the future. 

